# Interferon levels and interferon-stimulated gene expression identify patient subsets with distinct clinical and immunological characteristics in systemic lupus erythematosus

**DOI:** 10.3389/fimmu.2026.1757895

**Published:** 2026-01-29

**Authors:** Ridi Khatri, Amrutha Jose, Anjali Rajadhyaksha, Kalpana Mehta, Nilesh Nolkha, Seema Kini, Milind Nadkar, Pooja Jaiswal, Pratiksha Pawar, Swapnal Pawaskar, Kavyasree S, Ajit Kumar Das, Durga Chougule, Kirti Rai, Harshada Konkar, Seema Korgaonkar, Smruti Patil, Prathamesh Warang, Aman Malik, Manisha Madkaikar, Vandana D. Pradhan

**Affiliations:** 1 Department of Clinical and Experimental Immunology, ICMR-National Institute of Immunohaematology, Mumbai, Maharashtra, India; 2 ICMR-National Institute of Immunohaematology, Mumbai, Maharashtra, India; 3 Department of Medicine, Seth Gordhandas Sunderdas (G.S.) Medical College and King Edward Memorial Hospital, Mumbai, Maharashtra,, India; 4 Department of Nephrology, Topiwala National Medical College (T.N.M.C.) & Bai Yamunabai Laxman Nair Charitable (B.Y.L.N.Ch.) Hospital, Mumbai, Maharashtra, India; 5 Department of Medicine and Rheumatology, Topiwala National Medical College (T.N.M.C.) & Bai Yamunabai Laxman Nair Charitable (B.Y.L.N.Ch.) Hospital, Mumbai, Maharashtra, India; 6 Department of Pediatric Immunology & Leukocyte Biology, ICMR-National Institute of Immunohaematology, Mumbai, Maharashtra, India

**Keywords:** autoantibodies, clinical manifestations, disease activity, interferons, interferon-stimulated gene (ISG) expression, patient stratification, systemic lupus erythematosus

## Abstract

**Introduction:**

Interferon(IFN) system is dysregulated in Systemic Lupus Erythematosus(SLE) and represents potential therapeutic target. However, most studies have focused on isolated IFN types, particularly type I and type II, while type III IFNs remain poorly characterized. Comprehensive analysis of different IFN types in parallel with interferon-stimulated gene(ISG) expression is limited despite the interconnectedness of IFN families and their potential to differentially influence disease activity, immune phenotypes, and treatment responses. The present study assessed levels of IFN types and IFN score to evaluate their association with disease activity, clinical manifestations, and autoantibody profile in SLE patients from Western India.

**Methods:**

This cross-sectional study included clinically diagnosed SLE patients(n=115). Serum IFNα and IFNλ1-λ4 levels were detected using ELISA, while IFNγ levels were detected using bead-based assay, and IFN score by RT-qPCR based on the expression of five ISGs. SLE patients with IFN levels above third quartile were categorized as ‘IFN high’ groups, and their association with clinical and autoantibody profile were analysed using logistic regression. To identify patient subgroups based on autoantibody profile, unsupervised clustering was employed.

**Results:**

SLE patients showed significantly elevated IFNα(p<0.001), IFNγ(p=0.009), and IFNλ3(p<0.001) levels as well as IFN score(p<0.001) as compared to healthy controls. IFN score(r=0.228;p=0.014) and IFNα levels(r=0.430;p<0.001) correlated positively with disease activity. IFNα high group was associated with leukopenia (OR(95%CI):5.81(1.29,26.20);p=0.022) and multiple autoantibodies, while IFNγ high group with rash (OR(95%CI):2.73(1.06,7.00);p=0.037). FNλ3 high group showed positive association with anti-Ro52 autoantibodies (OR(95%CI):2.64(1.07,6.52);p=0.035) and negative association with low complement (OR(95%CI):0.35(0.13,0.89);p=0.028). IFNλ4 levels were not significantly elevated in SLE patients(p=0.642), however the levels were significantly associated with IFN score(r=0.359,p<0.001) and anti-dsDNA positivity(r=0.323,p<0.001), with higher levels observed in IFN score high SLE patients(p=0.016). Autoantibody profile-based clustering identified three subgroups differing in IFNα levels, IFN scores, disease activity, and associated immunological parameters.

**Conclusion:**

All three IFN pathways were elevated in SLE. Correlation of IFNα levels and IFN score with SLEDAI suggested their potential as possible biomarker for monitoring disease activity. Association of IFNλ4 with IFN score suggested their possible role in IFN pathway activation.By assessing IFNs at both protein and transcriptional levels, present study provided comprehensive insight into IFN pathway dynamics and IFN-driven heterogeneity in SLE.

## 1 Introduction

Systemic Lupus Erythematosus (SLE) is a highly heterogeneous autoimmune disorder characterized by diverse clinical and immunological manifestations, predominantly affecting females of reproductive age. The plethora of autoantibodies due to loss of immune tolerance is a hallmark of SLE. Although the precise mechanisms underlying SLE pathogenesis remain elusive, evidences suggest that a complex interplay of genetic, environmental, and immunological factors drives disease development and progression (1–5). In particular, an altered cytokine milieu, involving interferons (IFNs) and other inflammatory cytokines, plays an important role in amplifying autoreactive immune responses and promoting inflammation (6–9).

IFNs are key signalling molecules that exert anti-viral, inflammatory, and immune regulatory functions (10, 11). There are three major types of IFNs according to their receptor specificities, structures, and biological activities: type I (IFNα, IFNβ, IFNϵ, IFNκ, and IFNω), type II (IFNγ), and type III (IFN-λ1/IL-29, -λ2/IL-28A, -λ3/IL-28B, and -λ4) (11–13). The persistent production of IFNs, driven by a positive feedback mechanism, leads to aberrant immune activation and disease exacerbation in SLE. The elevated levels of IFNs induce the expression of IFN-stimulated genes (ISGs), known as IFN signature (1, 11, 14, 15). Studies have reported elevated type I IFN activity, particularly IFNα levels and ISG expression, which correlated with disease activity, severity, and specific organ involvement in SLE patients (6–9, 11, 12, 14). While type I IFNs have been implicated in the pathogenesis of SLE, clinical trials targeting this pathway have shown efficacy only in subsets of patients, indicating the impact of disease heterogeneity and the limitation of ISG expression as a predictive biomarker (16). Emerging evidences have also suggested the role of type II and type III IFNs (10, 11, 17, 18); however, few studies have simultaneously assessed different IFN types in parallel with their transcriptional profile (16, 19). As a result, the interplay among different IFN types and their relative contribution to disease activity and clinical and immunological heterogeneity remains an area of ongoing investigation.

SLE is characterized by the presence of different autoantibodies that not only serve as diagnostic markers but are also linked to diverse disease phenotypes and organ involvement (20). Given the important role of IFNs in promoting autoantibody production through B cell activation (12, 14, 21), we hypothesized that autoantibody-based stratification may reveal biologically meaningful subgroups characterized by differential IFN pathway activation and associated immunological parameters. Such stratification may further help to characterize SLE heterogeneity and identify patients for possible personalized therapy in the future. The role of IFNs in SLE pathogenesis is well reported in the available literature; however, the findings across individual studies have often been inconsistent, and a comprehensive analysis of different IFN types is limited.

To address this gap, a cross-sectional study assessing serum levels of type I, II, and III IFNs in SLE patients from Western India was conducted, and their association with disease activity was examined. SLE patients were further stratified into IFN high groups based on IFN levels, and the clinical manifestations and autoantibody profile associated with these groups were systematically evaluated. Additionally, hierarchical clustering was performed to assess if variations in IFN levels and other laboratory parameters aligned with distinct autoantibody-defined patient subgroups.

## 2 Materials and methods

### 2.1 Patients and controls

In the present cross-sectional study, participants were recruited using consecutive sampling. The primary objective was to compare three types of IFN and the composite IFN score, encompassing a total of seven key parameters, all of which were considered equally important for analysis. Accordingly, clinically diagnosed adult SLE patients (n=115) who met the EULAR/ACR 2019 classification criteria were consecutively enrolled over a period of three-years (2022–2025) based on the availability of eligible cases referred to our institute (22). These patients were enrolled from the Department of Medicine, King Edward Memorial Hospital, Mumbai, India, and the Department of Medicine and Rheumatology, and the Department of Nephrology, BYLN Nair Hospital, Mumbai, India. Cases of overlap with other autoimmune disorders, paediatric SLE patients, pregnant and post-menopausal women, and cases with malignancy or chronic infections were not included in the study. Human research was conducted in accordance with the Declaration of Helsinki. Demographic, clinical, and laboratory data was recorded for each patient at enrollment. The Safety of Estrogens in Lupus Erythematosus National Assessment-Systemic Lupus Erythematosus Disease Activity Index (SELENA-SLEDAI) score was used to evaluate the disease activity (23). As a control group, age and sex matched healthy controls (HCs) (n=65) were also enrolled. The ethical approval for this study was granted by ethics committees for Research on Human Subjects of all collaborating institutions. All participants provided written informed consents. Blood samples were collected in plain and tempus tubes for serological and ISG expression analyses, respectively. After initial processing, these samples were aliquoted and stored at -80 °C until further analysis. A flow diagram outlining the patient recruitment process has been provided in the **Supplementary Figure S1**.

### 2.2 Assessment of serum autoantibodies, IFNs and other immunological parameters

Anti-nuclear antibodies (ANA) and anti-dsDNA autoantibodies were screened by indirect immunofluorescence assay (IFA) using HEp-2 cells and *Crithidia lucillae*, respectively (EUROIMMUN, Germany). ANA specificity was further assessed with a LINE blot assay (EUROIMMUN, Germany). Serum IFNα levels were quantified using a pan-IFNα ELISA kit (3425-1A-20, Mabtech AB, Nacka, Sweden), capable of detecting IFNα subtypes 1/13, 2, 4, 5, 6, 7, 8, 10, 14, 16, and 17. Serum IFNγ levels were quantified using Multiplex bead-based assay (AimPlex Biosciences Inc., California, USA), and IFNλ1-λ4 levels were quantified using commercially available ELISA Kits (SARD Biosciences, Maharashtra, India). IFNλ4 levels were detected in 113 (98.3%) SLE patients and 59 (92.8%) HCs. Since the IFN data did not follow a normal distribution, determining the cut-offs using mean + 2SD of healthy controls was not appropriate. Hence, based on the literature, we considered the cut-off using the third quartile (Q_3_) (19, 24, 25). Individuals with values≥Q_3_ were defined as the ‘IFN high’ group, while those with values<Q_3_ were defined as the ‘IFN low’ group. Complement components (C3 and C4) were quantified using a MISPA-i3 nephelometer (Agappe, Kerala, India), with reference ranges of 80–180 mg% for C3 and 10–40 mg% for C4.

### 2.3 Assessment of ISG expression

Total RNA was extracted from whole blood collected in Tempus RNA blood tubes (4342792, Applied Biosystems, Foster City, CA) using a commercially available RNA extraction kit (4380204, Applied Biosystems, Foster City, CA). RNA concentration was estimated using Qubit (Q10210, Thermo Fisher Scientific, USA), and 100ng of RNA was reverse-transcribed using the High-Capacity cDNA Reverse Transcription Kit (4368814, Applied Biosystems, Foster City, CA) according to the manufacturer’s instructions. cDNA was then subjected to quantitative real-time PCR using the PowerUp SYBR Green Master Mix for qPCR (A25742, Applied Biosystems, Foster City, CA). Specificity of the PCR products was verified through melt curve analysis, and a standard curve was prepared for each PCR experiment. The expression of five ISGs namely, IRF1, MX1, RSAD2, IFI44L, and IFIT1, was quantified in duplicate by RT-qPCR. These ISGs represent widely validated markers of type I IFN pathway activation in SLE and are consistently reported in the literature (26–29). Gene expression was normalized to β-actin as a housekeeping gene (HKG), and relative expression was calculated using the 2^−ΔΔCt method (26). For each ISG, the ΔCt value was calculated as ΔCt_ISG_= Ct_ISG_ - Ct_HKG_. A calibration value was obtained from the mean ΔCt of HCs (ΔCt_Calibration_= Mean ΔCt_HCs_). The ΔΔCt for each patient sample was then derived as ΔΔCt= ΔCt_SLE_ - ΔCt_Calibration_, and relative gene expression was computed as 2^-ΔΔCt. The IFN score for each individual was generated by taking the median relative expression of the five ISGs. As the IFN score data did not follow a normal distribution, the third quartile (Q_3_) cut-off was used to classify SLE patients into the ‘IFN score high’ group for subsequent analyses.

### 2.4 Statistical analysis

Statistical analyses were performed using IBM SPSS Statistics (version 27) and R (version 4.5.1). Normality of continuous variables was assessed using the Kolmogorov–Smirnov and Shapiro–Wilk tests. As most variables deviated from normality, non-parametric tests were applied. Categorical variables were summarized as frequencies and percentages, while continuous variables were reported as medians with first (Q1) and third (Q3) quartiles. Group comparisons for continuous variables were done using the Mann–Whitney U test, and associations between categorical variables were examined using the chi-square test. Correlations were assessed with Spearman’s rank correlation coefficient. Logistic regression models, adjusted for disease duration, were employed to estimate odds ratios (ORs) with 95% confidence intervals (CIs). To identify patient subgroups based on autoantibody profile, an unsupervised clustering approach was employed. Since all variables included in the clustering were categorical in nature, pairwise dissimilarities were computed using Gower’s distance metric, which is appropriate for mixed and categorical data. Hierarchical clustering was then performed on the resulting dissimilarity matrix using an agglomerative approach. Cluster solutions were evaluated based on dendrogram structure and clinical interpretability. Comparisons between clusters were conducted using the Kruskal-Wallis test with Dunn’s *post hoc* multiple comparisons. A p-value<0.05 was considered statistically significant.

## 3 Results

### 3.1 Demographic, clinical, and laboratory characteristics of SLE patients

Demographic, clinical, and laboratory characteristics of SLE patients (n=115) enrolled in this cross-sectional study are summarized in **Table 1**. The median age at enrolment was 28 years (Q_1_, Q_3_: 22, 36) and the median age of onset was 26 years (Q_1_, Q_3_: 20, 31). The cohort consisted predominantly of females (91.3%), with males representing 8.7%, resulting in a female:male ratio of 10.5:1. The median duration of treatment received was 3 months (Q_1_, Q_3_: 0, 19). The median SLEDAI score was 9 (Q_1_, Q_3_: 6, 12). Among clinical manifestations, musculoskeletal manifestations were the most common (54.8%), followed by alopecia, rash, and photosensitivity observed in 53.9%, 51.3%, and 45.2% patients, respectively. Other notable clinical manifestations included lupus nephritis (LN) (39.1%), mucosal ulcers (37.4%), and constitutional manifestations (32.2%). Pyuria (7.8%), hematuria (6.1%), and neuropsychiatric manifestations (4.3%) were observed at a lower frequency. A high prevalence was noted for ANA positivity (96.5%), and anti-dsDNA autoantibodies showed 63.5% positivity. Complement levels indicated that 55.7% and 51.3% of patients had low C3 and C4 levels, respectively, with 40.9% patients exhibiting reductions in both complement components. Haematological abnormalities included thrombocytopenia in 14.8% and leukopenia in 7.0% of patients. Detailed laboratory parameters of the study participants are provided in **Supplementary Table S1**. Regarding treatment, 80.9% of patients received hydroxychloroquine, while 89.6% were taking prednisone at the time of enrolment. Cyclophosphamide and azathioprine were received by 27.8% and 5.2% patients, respectively.

**Table 1:** Demographic, clinical, and laboratory characteristics of SLE patients (n=115).

Demographic characteristics	
Age at enrolment (years)	28 (22, 36)
Age of onset (years)	26 (20, 31)
Females	105 (91.3%)
Males	10 (8.7%)
Female: Male	10.5:1
Duration of treatment (months)	3 (0, 19)
SLEDAI score	9 (6, 12)

Clinical manifestations	
Photosensitivity	52 (45.2%)
Malar rash	32 (27.8%)
Alopecia	62 (53.9%)
Mucosal ulcers	43 (37.4%)
Rash	59 (51.3%)
Musculoskeletal	63 (54.8%)
Lupus Nephritis	45 (39.1%)
Proteinuria	25 (21.7%)
Pyuria	9 (7.8%)
Hematuria	7 (6.1%)
Haematological	20 (17.4%)
Constitutional	37 (32.2%)
Serositis (Plerutitis, Pericarditis)	9 (7.8%)
Neuropsychiatric	5 (4.3%)

Laboratory parameters	
ANA positivity	111 (96.5%)
Anti-dsDNA positivity	73 (63.5%)
Low C3 (<80 mg%)	64 (55.7%)
Low C4 (<10 mg%)	59 (51.3%)
Low C3 and C4	47 (40.9%)
Thrombocytopenia (<100 x 10^9/L)	17 (14.8%)
Leukopenia (<3 x 10^9/L)	8 (7.0%)

Treatment	
Hydroxycholoquine	93 (80.9%)
Prednisone	103 (89.6%)
Cyclophosphamide	32 (27.8%)
Azathioprine	6 (5.2%)

Continuous variables are presented as median (Q_1_, Q_3_) and categorial variables as frequency (%). SLEDAI, Systemic lupus erythematosus disease activity index; ANA, anti-nuclear antibody; C3, Complement C3; C4, Complement C4.

### 3.2 Comparison of circulating IFN levels and ISG expression among SLE patients and HCs

**Figure 1:**
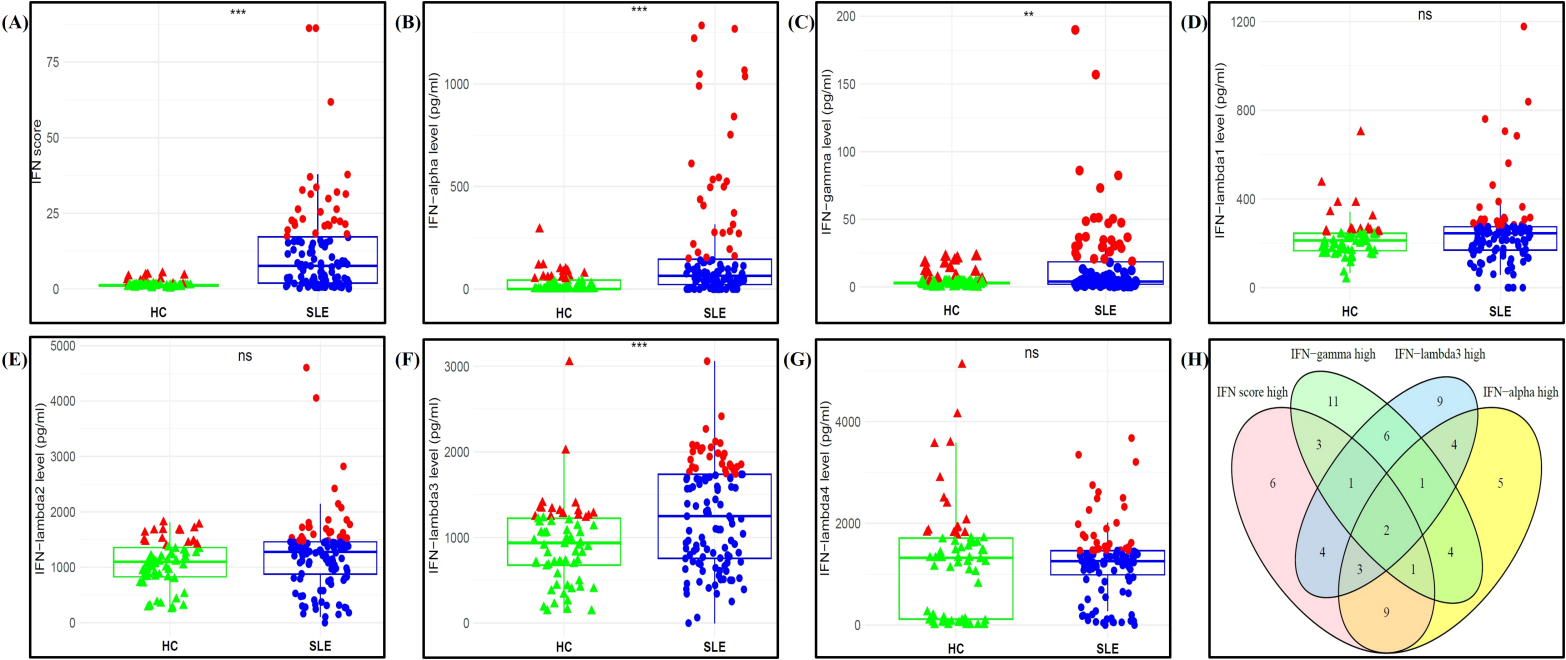
Comparison of **(A)** IFN score, **(B)** IFNα, **(C)** IFNγ, **(D)** IFNλ1, **(E)** IFNλ2, **(F)** IFNλ3, and **(G)** IFNλ4 levels between SLE patients (n=115) and HCs (n=65). **(H)** Venn diagram illustrating the distribution and overlap of individuals classified as ‘IFN high’ across the different IFN parameters. Each data point (dot) represents an individual participant. Box-and-whisker plots indicate the median and interquartile range (IQR) for each group. For each IFN parameter, values at or above the third quartile (Q3) of the overall distribution are highlighted in red and classified as the corresponding IFN high group, while values below Q3 are shown in green for HCs and blue for SLE patients. Between-group comparisons were performed using the Mann-Whitney U test. Statistical significance is denoted as **p < 0.01, and ***p < 0.001. ns, non significant.

The box plots illustrate the comparison of IFN score, and type I (IFNα), type II (IFNγ), and type III IFNs (IFNλ1-λ4), among SLE patients and HCs (**Figures 1A–G**). IFN score was significantly higher in SLE patients as compared to HCs (p<0.001). IFN levels and Individual RT-qPCR expression data for all five ISGs (IRF1, MX1, RSAD2, IFI44L, and IFIT1) are provided in the **Supplementary Material** (**Supplementary Table S2**). All five ISGs were significantly elevated in SLE patients as compared to HCs (all p<0.05). Similarly, significantly elevated serum levels of IFNα (p<0.001), IFNγ (p=0.009), and IFNλ3 (p<0.001) were observed in SLE patients as compared to HCs. SLE patients with IFN score and IFN levels above the third quartile (Q_3_) were categorized in ‘IFN high’ groups. It was observed that 29 patients (25.2%) were categorized in IFN score high, IFNα high, and IFNγ high groups, and 30 patients (26.1%) were in IFNλ3 high group. A Venn diagram (**Figure 1H**) illustrates the distribution and overlap among these ‘IFN high’ groups.

### 3.3 Correlation between IFN score, IFN levels with disease activity and immunological parameters among SLE patients

**Figure 2:**
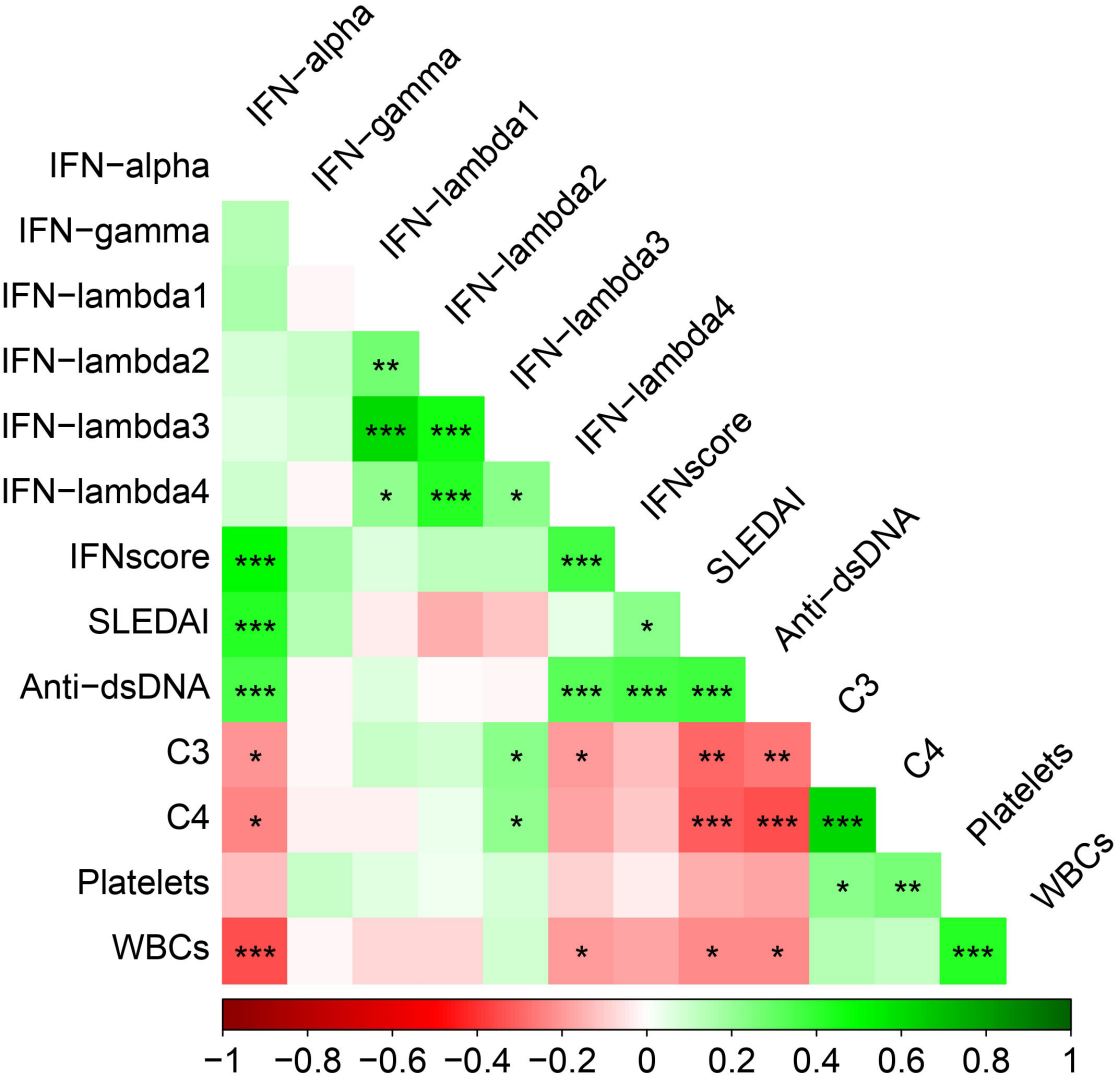
Correlation matrix depicting the associations between circulating IFN levels, IFN score, disease activity, and other immunological parameters among SLE patients (n=115). Correlations were assessed using Spearman’s rank correlation for continuous variables and point-biserial correlation for associations between continuous and binary variables. Each cell represents the corresponding correlation coefficient between the variables indicated on the x- and y-axes. The color gradient reflects the strength and direction of the association, with green indicating positive correlations, red indicating negative correlations, and white indicating no linear correlation. Statistical significance is denoted as *p < 0.05, **p < 0.01, and ***p < 0.001.

The correlation between IFN score, IFN levels, disease activity, and other immunological parameters among SLE patients is illustrated in **Figure 2**. The SLEDAI score showed a significant positive correlation with IFN score (r= 0.228; p=0.014) and IFNα levels (r= 0.430; p<0.001). Anti-dsDNA autoantibodies were positively correlated with IFN score, IFNα, and IFNλ4 (all p<0.001). Complement component C3 was negatively correlated with IFNα (r=-0.204; p=0.029) and IFNλ4 (r=-0.201; p=0.031) levels, but positively correlated with IFNλ3 (r=0.224; p=0.016). Similarly, C4 levels were negatively correlated with IFNα (r=-0.230, p=0.013) and positively correlated with IFNλ3 levels (r=0.220; p=0.018). Additionally, IFNα (r=-0.345; p<0.001) and IFNλ4 (r=-0.195; p=0.037) levels were negatively correlated with WBCs. Notably, serum levels of IFNα (r=0.509; p<0.001) and IFNλ4 (r=360; p<0.001) were positively correlated with IFN score. The correlation between serum IFNλ4 levels and IFN score is shown in **Supplementary Figure S2A**, while comparisons of IFNλ4 levels between anti-dsDNA positive and anti-dsDNA negative SLE patients and between IFN score high and IFN score low groups are presented in **Supplementary Figures S2B** and **S2C**, respectively.

### 3.4 Association between IFN high groups and clinical manifestations among SLE patients

**Figure 3:**
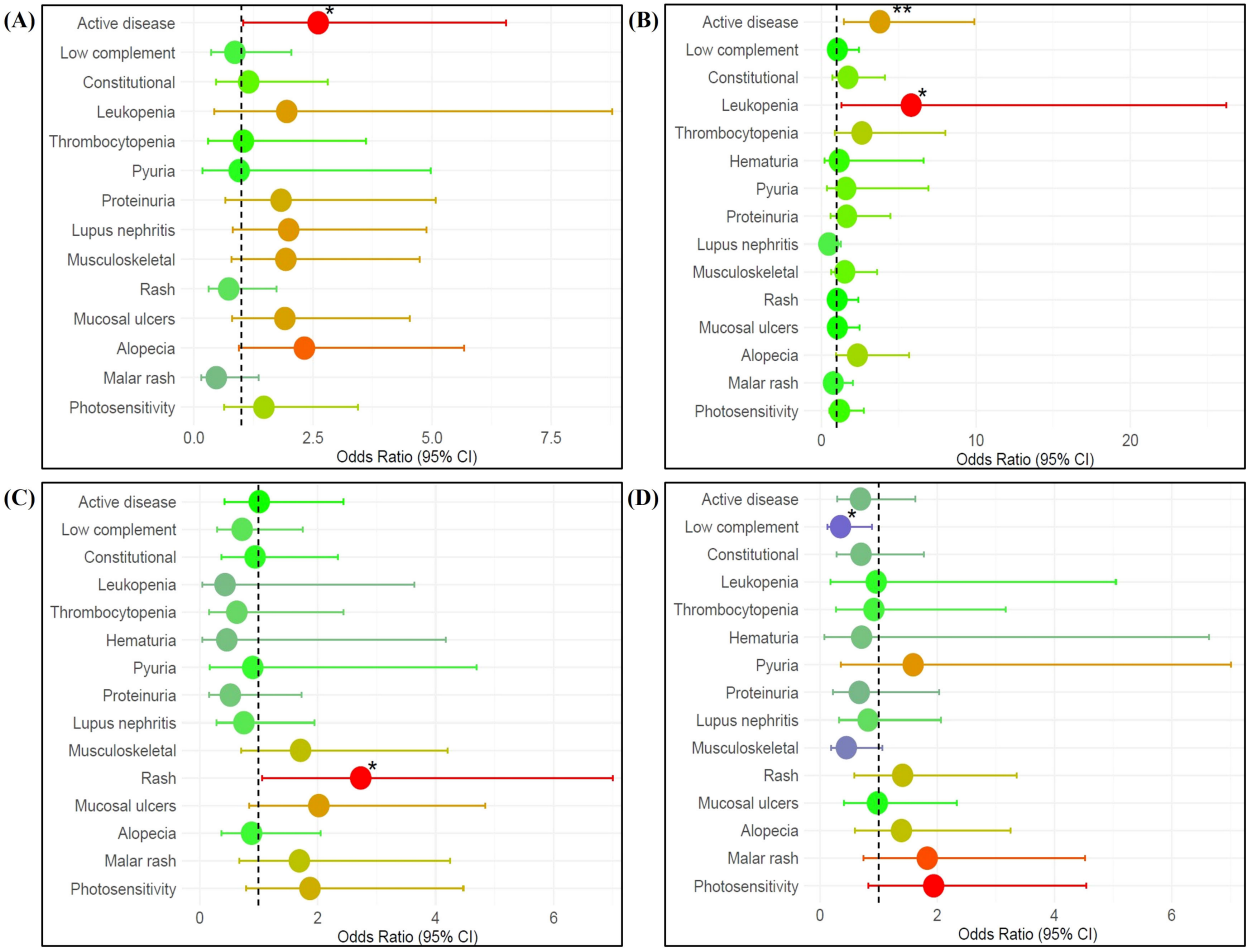
Association between **(A)** IFN score high, **(B)** IFNα high, **(C)** IFNγ high, and **(D)** IFNλ3 high groups with clinical manifestations among SLE patients (n=115). Multivariable logistic regression, adjusted for disease duration, was used to estimate odds ratios (ORs) and 95% confidence intervals (CIs). Each point represents the OR for a specific clinical manifestation in IFN high patients. ORs>1 indicate increased odds of the clinical manifestation in IFN high patients, whereas ORs<1 indicate reduced odds relative to IFN low patients. The dashed vertical line at OR = 1 represents the null association, and horizontal bars denote 95% CIs. Point estimates are color-coded according to the magnitude of the OR (red: OR>1; green: OR≈1; blue: OR<1). Statistical significance is denoted as *p < 0.05, **p < 0.01.

**Figure 3** illustrates an association between IFN high groups with clinical manifestations among SLE patients. It was observed that high IFN score and IFNα levels were associated with active disease. High IFNα levels were positively associated with leukopenia (OR (95% CI): 5.81 (1.29, 26.20); p=0.022), while high IFNγ levels were positively associated with rash (OR (95% CI): 2.73 (1.06, 7.00); p=0.037). High IFNλ3 levels were negatively associated with low complement (OR (95% CI): 0.35 (0.13, 0.89; p=0.028).

### 3.5 Association between IFN high groups and autoantibodies among SLE patients

**Figure 4:**
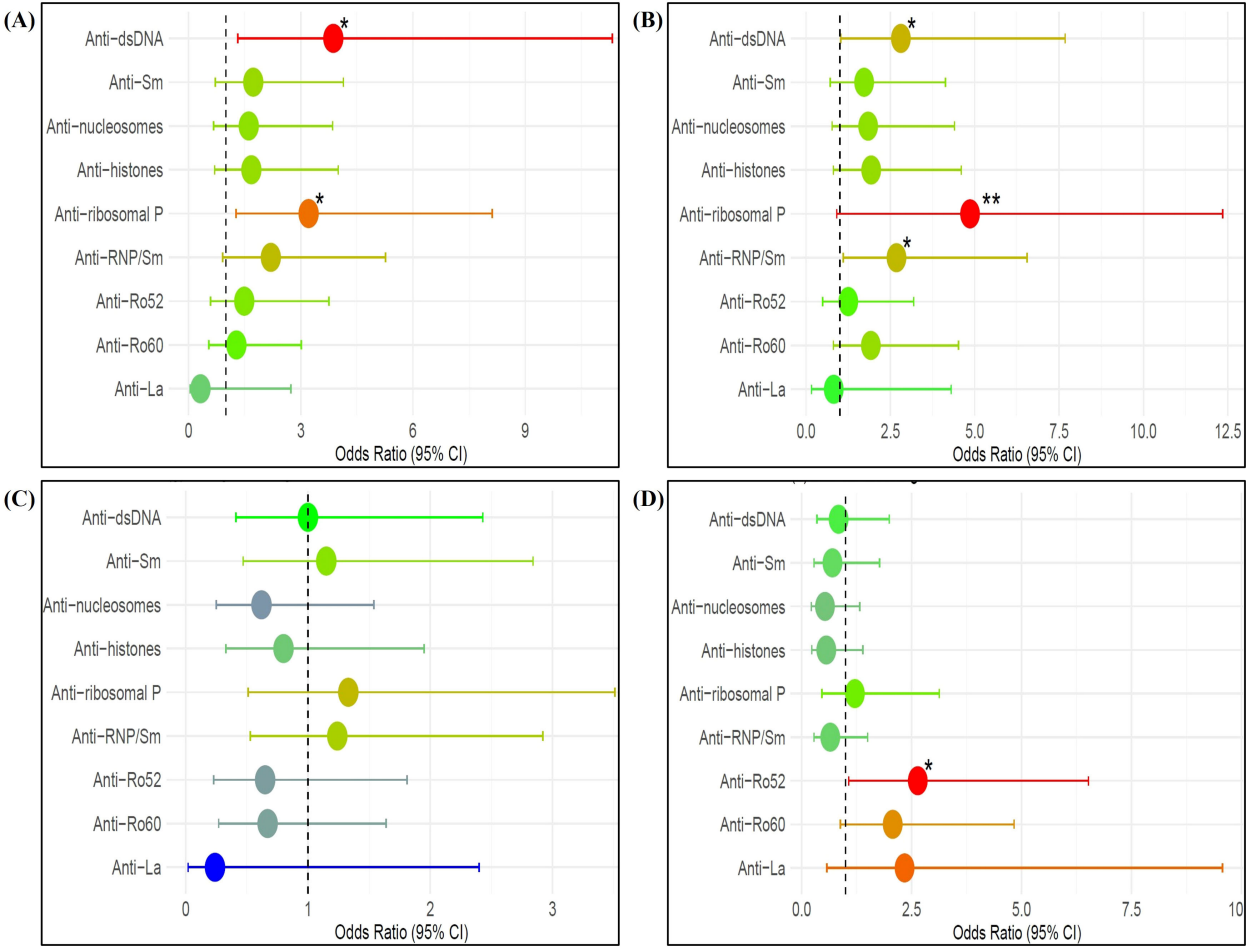
Association between **(A)** IFN score high, **(B)** IFNα high, **(C)** IFNγ high, and **(D)** IFNλ3 high groups with autoantibodies among SLE patients (n=115). Multivariable logistic regression, adjusted for disease duration, was used to estimate odds ratios (ORs) and 95% confidence intervals (CIs). Each point represents the OR for a specific autoantibody positivity in IFN high patients. ORs>1 indicate increased odds of autoantibody positivity in IFN high patients, whereas ORs<1 indicate reduced odds relative to IFN low patients. The dashed vertical line at OR = 1 represents the null association, and horizontal bars denote 95% CIs. Point estimates are color-coded according to the magnitude of the OR (red: OR>1; green: OR≈1; blue: OR<1). Statistical significance is denoted as *p < 0.05, **p < 0.01.

An association between IFN high groups with autoantibodies is illustrated in **Figure 4**. High IFN score was observed to be associated with anti-dsDNA autoantibodies (OR (95% CI): 3.87 (1.32, 11.33); p=0.014) and anti-ribosomal P autoantibodies (OR (95% CI): 3.21 (1.27, 8.12); p=0.014), while high IFNα levels were observed to be associated with anti-dsDNA autoantibodies (OR (95% CI): 2.81 (1.03, 7.68); p=0.044), anti-ribosomal P autoantibodies (OR (95% CI): 4.86 (0.91, 12.35); p=0.001), and anti-RNP/Sm autoantibodies (OR (95% CI): 2.68 (1.10, 6.55); p=0.031). High IFNγ levels were not associated with any autoantibodies. High IFNλ3 levels were positively associated with anti-Ro52 autoantibodies (OR (95% CI): 2.64 (1.07, 6.52); p=0.035).

### 3.6 Cluster analysis based on autoantibody profile in SLE patients and comparison of IFN levels and immunological parameters among identified clusters

**Figure 5:**
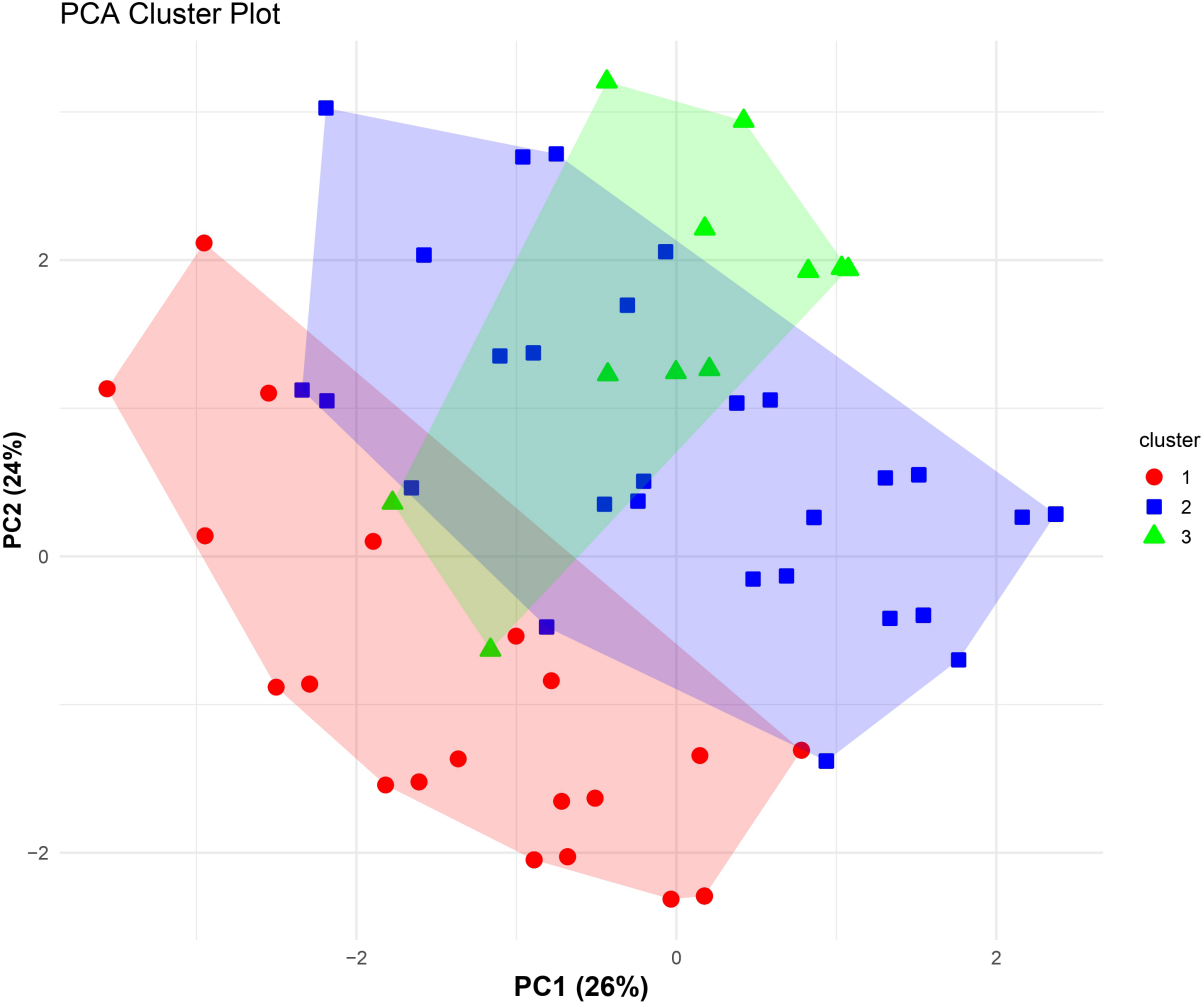
PCA-based cluster plot illustrating patient subgroups identified through hierarchical clustering of SLE patients (n=115) based on autoantibody profile. Unsupervised agglomerative hierarchical clustering was performed using Gower’s distance to accommodate categorical autoantibody variables. Patients are projected onto the first two principal components (PC1 and PC2), which together explain 50% of the total variance (PC1: 26%, PC2: 24%). Colored symbols represent cluster membership, and shaded polygons indicate the dispersion of each cluster in PCA space, demonstrating separation of immunologically distinct autoantibody-defined subgroups within the SLE cohort.

Hierarchical clustering of SLE patients based on their autoantibody profile revealed three distinct clusters, as illustrated in the cluster plot in **Figure 5**. The optimum number of clusters were estimated by both elbow and silhouette methods. **Table 2** represents the comparison of IFN levels along with various clinical and immunological parameters within the identified clusters. A significant difference in IFN score (p=0.049) and IFNα levels (p=0.015) was observed among these clusters. Disease activity, assessed by SLEDAI score, also varied significantly across these clusters (p<0.001). Among laboratory parameters, WBCs (p=0.027), RBCs (p=0.002), and complement C3 (p=0.026) and C4 (p=0.016) levels demonstrated significant differences among these autoantibody-based clusters.

**Table 2:** Comparison of IFN levels and various immunological parameters among the identified clusters (n=115).

Parameters	Cluster 1 n=43	Cluster 2 n=57	Cluster 3 n=15	P-value
IFN Score	9.06 (3.20, 19.43)	7.62 (2.17, 17.70)	2.19 (1.07, 8.17)	**0.049**
IFNα (pg/ml)	98.16 (41.83, 274.11)	43.96 (3.38, 110.49)	71.00 (41.38, 100.79)	**0.015**
IFNγ (pg/ml)	3.49 (2.24, 10.82)	4.66 (1.73, 27.77)	4.81 (1.66, 10.52)	0.954
IFNλ1 (pg/ml)	248.49 (133.92, 283.38)	239.29 (174.36, 274.90)	257.48 (150.00, 274.90)	0.980
IFNλ2 (pg/ml)	1149.13 (580.03, 1438.62)	1330.20 (961.88, 1474.01)	1262.48 (581.74, 1544.73)	0.339
IFNλ3 (pg/ml)	936.69 (700.33, 1622.97)	1384.78 (833.36, 1745.58)	1573.03 (607.99, 1980.46)	0.184
IFNλ4 (pg/ml)	1311.42 (941.32, 1546.42)	1250.06 (1054.80, 1393.68)	1228.90 (208.82, 1303.62)	0.291
Age at enrolment (years)	27 (22, 34)	29 (21, 38)	30 (22, 39)	0.736
Age of onset (years)	25 (20, 31)	27 (19, 31)	29 (21, 33)	0.888
Treatment duration (months)	0.00 (0.00, 12.00)	7.00 (0.00, 26.00)	6.00 (0.00, 39.00)	0.076
SLEDAI	11 (8, 14)	6 (4, 10)	10 (7, 14)	**<0.001**
WBCs (x 10^9/L)	7.00 (4.00, 8.00)	8.00 (6.00, 10.50)	8.00 (6.00, 10.00)	**0.027**
RBCs (x 10^9/L)	3.00 (3.00, 4.00)	4.00 (3.50, 4.00)	4.00 (3.00, 4.00)	**0.002**
Platelets (x 10^9/L)	198.00 (99.00, 295.00)	258.00 (185.50, 340.50)	222.00 (132.00, 333.00)	0.116
C3 (mg%)	49.00 (36.00, 82.65)	88.93 (43.37, 120.15)	63.00 (32.68, 118.00)	**0.026**
C4 (mg%)	7.00 (5.30, 10.19)	13.07 (5.00, 26.11)	13.00 (8.00, 18.60)	**0.016**

Group comparisons were performed using the Kruskal-Wallis test followed by Dunn’s multiple comparisons test. p<0.05 was considered statistically significant. IFN, Interferon; SLEDAI, Systemic lupus erythematosus disease activity index; WBCs, White blood cells; RBCs, Red blood cells; C3, Complement C3; C4, Complement C4. Bold values represent statistically significant findings.

## 4 Discussion

Type I IFNs are the key modulators of immune responses and their dysregulation had been the focus of extensive research in SLE, with implications for assessing disease activity and therapeutic management. A significantly elevated type I IFN activity noted among SLE patients in the present study, as demonstrated by both elevated IFN score and serum IFNα levels, is consistent with previously published reports (19, 30–36). In addition, the elevated IFNγ and IFNλ3 levels among SLE patients suggested contribution of both type II and type III IFNs, which is in line with previous reports (19, 32, 37–48). Some studies had reported elevated IFNλ1 levels in SLE patients (32, 44, 46, 48); however, this was not observed in the present study. This may be attributed to the differences in disease activity, treatment modalities, or methodological approaches.

The correlation analyses of IFNs with disease activity and various laboratory parameters suggested their clinical significance and possible role in driving immunological dysregulation in SLE. A positive correlation between IFNα levels and IFN score with SLEDAI suggested their association with disease activity and supported their utility as potential biomarkers for disease monitoring. This finding was consistent with previous reports (19, 34, 36, 49–51). A strong positive association between IFNα levels and IFN score with anti-dsDNA antibodies had supported the role of type I IFNs in production of autoantibodies. In the present study, SLE patients did not show significantly elevated IFNλ4 levels as compared to HCs. However, IFNλ4 levels were significantly correlated with IFN score, an effect that was driven entirely by SLE patients and absent in HCs. Additionally, IFNλ4 levels were significantly higher in SLE patients with a high IFN score compared with those with a low IFN score. These findings suggested that IFNλ4 is linked to IFN pathway activation rather than to disease presence alone, reflecting the heterogeneous nature of IFN responses in SLE in which only a subset of patients exhibit high IFN activity. This pattern is consistent with previous reports showing that genetic variation within the IFNL3/IFNL4 locus influences the induction of ISGs in SLE and hepatitis C virus (HCV) infection (52–54). Notably, the association between IFNλ4 and anti-dsDNA autoantibodies has not been previously reported in SLE. However, previous studies had reported that elevated IFNλ1 levels were associated with anti-dsDNA autoantibodies (32, 48) and anti-nucleosome autoantibodies in SLE patients (19, 44). A study by Barnas et al. had reported that IFNλ1 promoted TLR7/8-mediated differentiation of human B cells into antibody-secreting plasma cells, suggesting a role of III IFNs in B-cell activation and production of autoantibodies (55). However, further mechanistic studies are required to understand the role of individual IFNλ types in autoantibody production (18, 55). A significant negative correlation between IFNα with both C3 and C4, and IFNλ4 with C3 suggested complement consumption. Interestingly, IFNλ3 indicated a positive correlation with both C3 and C4 levels, which suggested its possible regulatory role in maintaining complement homeostasis. However, further mechanistic studies are required to support these findings.

Patient stratification based on IFN levels revealed distinct clinical and autoantibody profile within the SLE cohort. Logistic regression analyses, adjusted for disease duration, demonstrated that IFN score high groups exhibited associations with anti-dsDNA autoantibodies and anti-ribosomal P autoantibodies. Similarly, IFNα high patients exhibited significant associations with leukopenia and multiple pathogenic autoantibodies, including anti-dsDNA, anti-ribosomal P, and anti-RNP/Sm autoantibodies. These findings supported an important role of type I IFNs in promoting B cell activation and production of autoantibodies (12, 14, 21). These findings aligned with a previous study by Gómez-Bañuelos et al. that had reported associations between high type I IFN and anti-dsDNA, anti-RNP, anti-Sm, and anti-Ro52 autoantibodies (16). Another study by Oke et al. had reported high IFNα levels were associated with mucocutaneous manifestations and anti-Ro52 and anti-La autoantibodies (19).

The IFNγ high patients were significantly associated with rash in the present study, suggesting their role in cutaneous manifestations in SLE (10). These findings differed from previous studies, where wither no associations with clinical manifestations (16) or associations with arthritis, low complement and anti-Ro60 autoantibodies (19) for elevated IFNγ levels had been reported. Notably, the IFNλ3 high group showed a negative association with low complement, which suggested a regulatory role, and a novel association with anti-Ro52 autoantibodies, which indicated a possible contribution of type III IFNs to specific autoantibody production. These findings differed from a previous study which had reported that high IFNλ3 levels were associated with fever, photosensitivity, musculoskeletal damage and anti-nucleosomes autoantibodies (19). Importantly, the heterogeneous distribution and limited overlap among IFN score, IFNα, IFNγ, and IFNλ3 high groups demonstrated the presence of distinct immunological endotypes within the SLE cohort. Thus, different patients may exhibited predominant activation of specific IFN pathways, which supported the concept of precision medicine, where therapeutic strategies can be tailored based on individual IFN profile and related immunological feature.

Hierarchical clustering based on autoantibody profile was performed to identify distinct patient subgroups within the SLE cohort that may represent immunophenotypic endotypes. This analysis identified three clusters, each characterized by significant differences in IFN levels, disease activity, and laboratory parameters. Cluster 1 exhibited the highest IFN score and IFNα levels, coupled with elevated disease activity and hypocomplementemia. In contrast, cluster 2 exhibited lowest IFNα levels and SLEDAI score, whereas cluster 3 exhibited lowest IFN score and elevated SLEDAI score. A significant difference in IFNα levels and IFN score among clusters suggested that IFN dysregulation was not uniform across SLE patients but was enriched in specific immunological subsets, indicating differential activation of IFN pathway. Similarly, a study by Kaan et al. had reported that autoantibody-defined clusters showed significant differences in IFN scores (20). The observed variation in SLEDAI scores among clusters further supported the clinical relevance of this stratification, linking autoantibody-defined groups with differential disease activity. Laboratory parameters including leukocyte and erythrocyte counts, as well as complement components C3 and C4, also varied significantly across clusters. While haematological differences achieved statistical significance, modest median shifts suggested that these findings should be interpreted cautiously from a clinical perspective.

To the best of our knowledge, this is the first study from India to measure different IFN types along with comprehensive clinical and laboratory profiling in a well characterized SLE cohort. The simultaneous assessment of circulating IFN levels and ISG expression provided insight into both protein and transcriptional level activation of the IFN pathway. The evaluation of different IFN types helped to understand their interplay and associations with various clinical manifestations and autoantibody profile in SLE. However, the cross-sectional design provided single time point data, limiting the assessment of changes in IFN levels with disease progression, flares, or treatment. Longitudinal studies are further required to understand IFN dynamics and their relevance to treatment response.

In conclusion, this study demonstrated that all three IFN pathways were elevated in SLE patients from Western India. The elevated IFN score, and IFNα, IFNγ, and IFNλ3 levels and their associations with various clinical and immunological parameters suggested their contribution to immune dysregulation in SLE. The correlation between IFNα levels and IFN score with disease activity suggested their potential utility as possible biomarkers for monitoring SLE disease activity. A comprehensive analysis of associations between IFN high groups and distinct clinical and autoantibody profile provided insight with implications for both diagnosis and personalized treatment strategies. Additionally, hierarchical clustering based on autoantibody profile revealed distinct immunophenotypic endotypes within the SLE cohort, which reflected SLE heterogeneity and supported the potential for more precise patient stratification. Future mechanistic and longitudinal investigations are essential to validate these observations and delineate causal links.

## Data Availability

The raw data supporting the conclusions of this article will be made available by the authors, without undue reservation.
