# Supplementary material for: Interferon levels and interferon-stimulated gene expression identify patient subsets with distinct clinical and immunological characteristics in systemic lupus erythematosus
Source: Front Immunol. 2026 Jan 29;17:1757895. doi: 10.3389/fimmu.2026.1757895 (PMC12894387; doi:10.3389/fimmu.2026.1757895)
Supplement: Supplementary file 1 [file Table1.docx]

**Supplementary Table S1 Laboratory parameters of SLE patients (n=115)**

| **Laboratory parameters** | |
| --- | --- |
| ANA positivity | 111 (96.5%) |
| Anti-dsDNA positivity | 73 (63.5%) |
| C3 (mg/dL) | 71.99 (37.00, 106.38) |
| Low C3 (<80 mg%) | 64 (55.7%) |
| C4 (mg/dL) | 9.63 (5.51, 21.17) |
| Low C4 (<10 mg%) | 59 (51.3%) |
| Low C3 and C4 | 47 (40.9%) |
| Platelets (<100 x 10^9/L) | 249.50 (143.00, 312.00) |
| Thrombocytopenia (<100 x 10^9/L) | 17 (14.8%) |
| WBCs (<3 x 10^9/L) | 7.00 (6.00, 10.00) |
| Leukopenia (<3 x 10^9/L) | 8 (7.0%) |

Continuous variables are presented as median (Q_1_, Q_3_) and categorial variables as frequency (%). ANA, anti-nuclear antibody; C3, Complement C3; C4, Complement C4; WBC, White blood cells
