# Supplementary material for: Interferon levels and interferon-stimulated gene expression identify patient subsets with distinct clinical and immunological characteristics in systemic lupus erythematosus
Source: Front Immunol. 2026 Jan 29;17:1757895. doi: 10.3389/fimmu.2026.1757895 (PMC12894387; doi:10.3389/fimmu.2026.1757895)
Supplement: Supplementary file 2 [file Table2.docx]

**Supplementary Table S2 Comparison of interferon levels and interferon-stimulated genes (ISGs) expression between SLE patients and healthy controls**

|  | **SLE (n=115)** | **HCs (n=65)** | **p-value** |
| --- | --- | --- | --- |
| **IFNα (pg/mL)** | 64.60 (22.62, 149.89) | 0.00 (0.00, 45.98) | **<0.001** |
| **IFNγ (pg/mL)** | 4.03 (2.02, 18.92) | 3.18 (2.36, 3.50) | **0.009** |
| **IFNλ1 (pg/mL)** | 245.12 (167.31, 274.90) | 212.59 (165.84, 247.39) | 0.079 |
| **IFNλ2 (pg/mL)** | 1281.75 (874.31, 1459.89) | 1102.00 (828.57, 1371.81) | 0.069 |
| **IFNλ3 (pg/mL)** | 1248.94 (755.12, 1737.84) | 936.52 (670.35, 1227.13) | **<0.001** |
| **IFNλ4 (pg/mL)** | 1257.14 (945.72, 1465.95) | 1320.00 (104.73, 1708.40) | 0.642 |
| **MX1** | 4.06 (1.21, 11.39) | 1.24 (0.46, 2.46) | **<0.001** |
| **IRF1** | 1.30 (0.97, 1.69) | 1.07 (0.81, 1.27) | **<0.001** |
| **RSAD2** | 6.82 (1.87, 18.51) | 1.16 (0.49, 2.23) | **<0.001** |
| **IFI44L** | 7.73 (2.03, 21.86) | 0.91 (0.42, 2.12) | **<0.001** |
| **IFIT1** | 12.13 (1.67, 43.71) | 1.13 (0.36, 4.29) | **<0.001** |
| **IFN score** | 6.50 (1.57, 14.22) | 1.07 (0.80, 1.26) | **<0.001** |

Data are represented as median (Q_1_, Q_3_). Group comparisons were performed using the Mann-Whitney U test. p<0.05 was considered statistically significant
