# Supplementary figures and images for: Interferon levels and interferon-stimulated gene expression identify patient subsets with distinct clinical and immunological characteristics in systemic lupus erythematosus
Source: Front Immunol. 2026 Jan 29;17:1757895. doi: 10.3389/fimmu.2026.1757895 (PMC12894387; doi:10.3389/fimmu.2026.1757895)

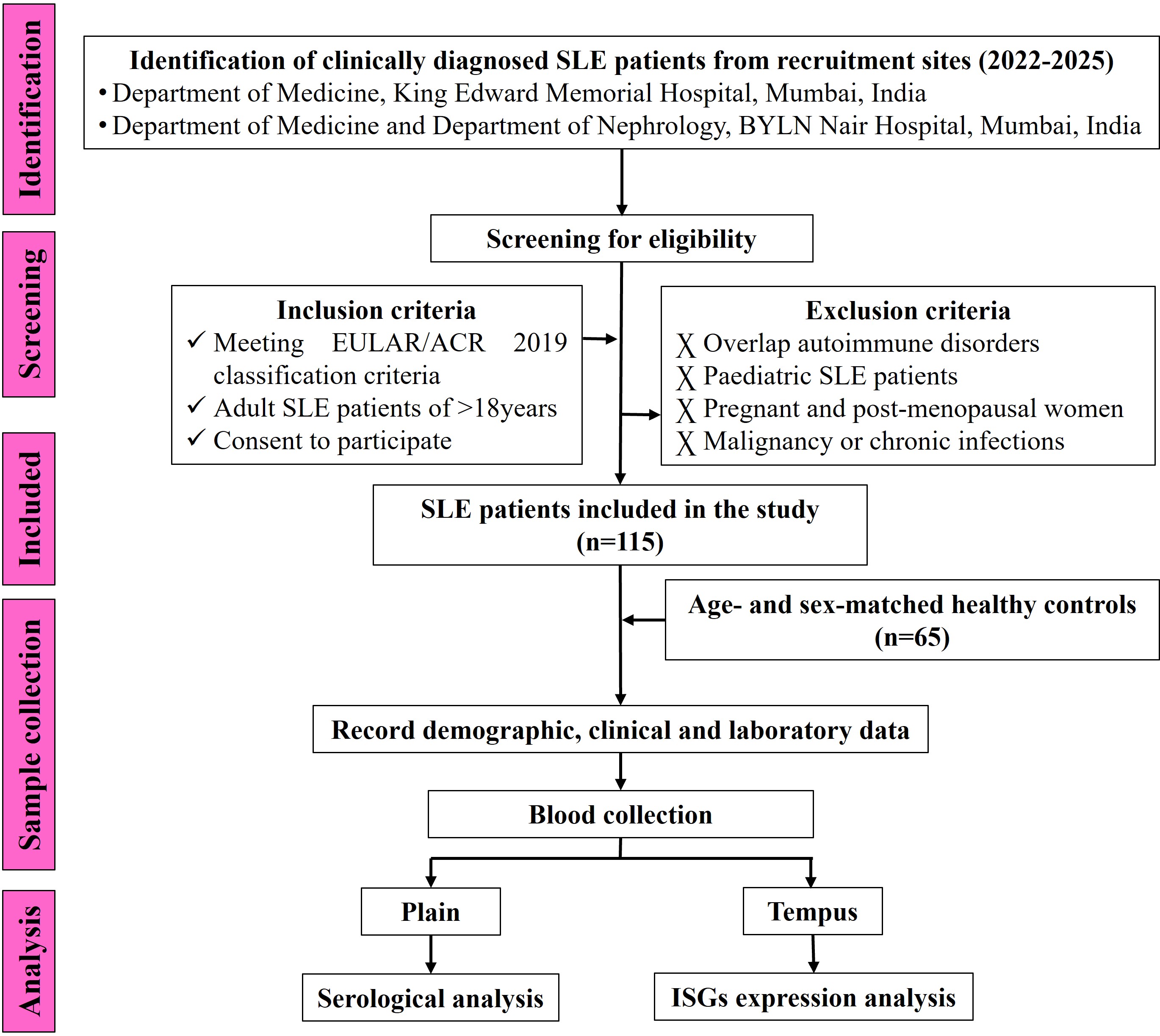

Supplement: Supplementary file 3 [file Image1.jpg]

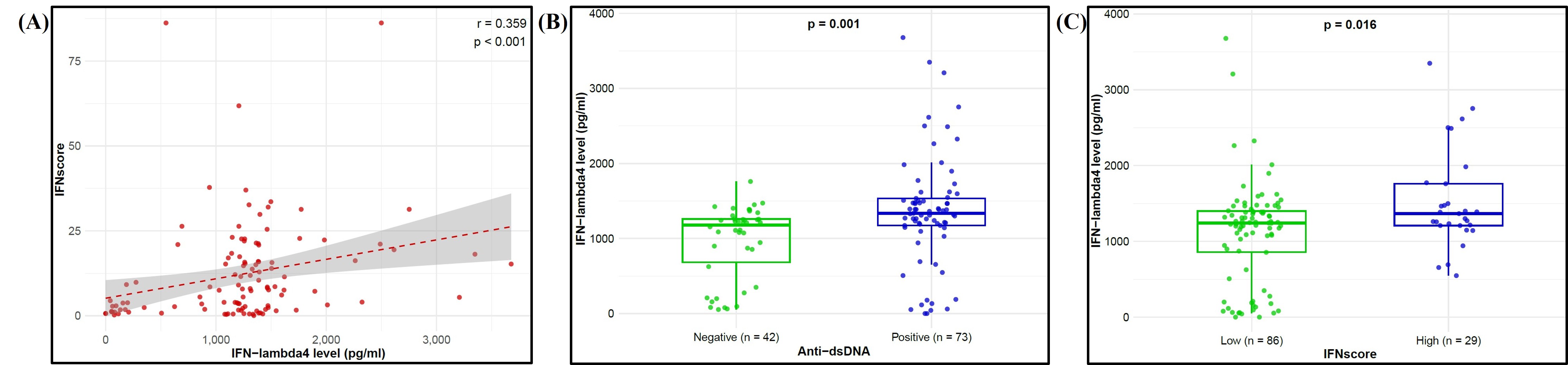

Supplement: Supplementary file 4 [file Image2.jpg]
